# Characteristics of phase-change materials containing oxide nano-additives for thermal storage

**DOI:** 10.1186/1556-276X-7-611

**Published:** 2012-11-06

**Authors:** Tun-Ping Teng, Chao-Chieh Yu

**Affiliations:** 1 Department of Industrial Education, National Taiwan Normal University, No. 162, Sec. 1, He-ping E. Rd., Taipei City, Da-an District, 10610, Taiwan

**Keywords:** Alumina, Nanocomposite-enhanced phase-change materials, Paraffin, Silica, Titania, Zinc oxide

## Abstract

In this study, the authors report the production of nanocomposite-enhanced phase-change materials (NEPCMs) using the direct-synthesis method by mixing paraffin with alumina (Al_2_O_3_), titania (TiO_2_), silica (SiO_2_), and zinc oxide (ZnO) as the experimental samples. Al_2_O_3_, TiO_2_, SiO_2_, and ZnO were dispersed into three concentrations of 1.0, 2.0, and 3.0 wt.%. Through heat conduction and differential scanning calorimeter experiments to evaluate the effects of varying concentrations of the nano-additives on the heat conduction performance and thermal storage characteristics of NEPCMs, their feasibility for use in thermal storage was determined. The experimental results demonstrate that TiO_2_ is more effective than the other additives in enhancing both the heat conduction and thermal storage performance of paraffin for most of the experimental parameters. Furthermore, TiO_2_ reduces the melting onset temperature and increases the solidification onset temperature of paraffin. This allows the phase-change heat to be applicable to a wider temperature range, and the highest decreased ratio of phase-change heat is only 0.46%, compared to that of paraffin. Therefore, this study demonstrates that TiO_2_, added to paraffin to form NEPCMs, has significant potential for enhancing the thermal storage characteristics of paraffin.

## Background

Thermal energy storage (TES) by solar power has become a popular research topic in recent years. Because of the impact of day and night on solar thermal energy storage, thus, the development of efficient energy storage materials will directly influence the utilization efficiency of solar thermal energy storage [1-3]. In general, single-phase thermal energy storage materials require a large storage space, which reduces the usefulness of thermal storage [4,5]. Therefore, developmental research on thermal energy storage materials focuses on phase-change materials (PCMs), and several research results and practical applications have been published [6-10].

Most PCMs have low thermal conductivity, which prevents them from overcoming problems of rapid load changes in the charging and discharging processes [11]. To overcome this obstacle and to obtain excellent thermal properties, studies have proposed various techniques for enhancing the thermal conductivity of PCMs, such as adding metallic or nonmetallic particles with high thermal conductivity [12-15], inserting fins [16-18], incorporating porous or expanded materials [19-27], inserting fibrous materials [28-31], and incorporating macro-, micro-, and nano-capsules [32-34]. The abovementioned methods for enhancing the thermal conductivity of PCM involve adding high-conductivity materials to improve the thermal conductivity of PCMs as the most simple and feasible method.

Metals have excellent thermal conductivity; therefore, they can be expected to enhance the thermal conductivity of PCMs significantly. However, metal materials oxidize, and their application to PCMs can degenerate and reduce the thermal conductivity of PCMs in the long run. Although adding metal oxides or minerals to PCMs to enhance thermal conductivity is worth considering, the thermal conductivity of such additives must be higher than that of the PCM if they are to enhance the thermal conductivity of PCMs. Moreover, a poor combination of additives to PCMs can increase interface thermal resistance and sedimentation and reduce the performance of the thermal storage without enhancing the thermal conductivity of PCMs. With the development of nanotechnology, the size of the additives can be reduced to a nanometer scale, and the reduced size can enhance the suspension performance, specific surface area, and heat transfer performance of the additives. In previous studies, carbon nanotubes (CNTs) [35-37], carbon nanofibers (CNFs) [37,38], Al_2_O_3_ nanoparticles [39-41], and Ag nanoparticles [42] were added to form nanocomposite-enhanced phase-change materials (NEPCMs) as a technique to enhance the thermal performance of PCMs. However, CNTs, CNFs, and Ag are expensive; therefore, the extensive use of these nanoparticles can reduce the economic benefits of PCMs for TES.

Paraffin is a material of low cost and toxicity that can be decomposed by bacteria. Therefore, it was chosen as the PCM thermal storage material for this study. Furthermore, the melting point of paraffin is approximately 55°C to 65°C, which makes it suitable for thermal storage in non-concentrating solar collectors. The direct-synthesis method was used to produce NEPCMs by adding alumina (Al_2_O_3_), titania (TiO_2_), silica (SiO_2_), and zinc oxide (ZnO) to paraffin. To demonstrate the feasibility of using NEPCMs in thermal storage systems, both the heat conduction and differential scanning calorimeter (DSC) experiments were used to assess the characteristics of NEPCMs and paraffin.

## Methods

### Preparation of NEPCMs

**Figure 1:**
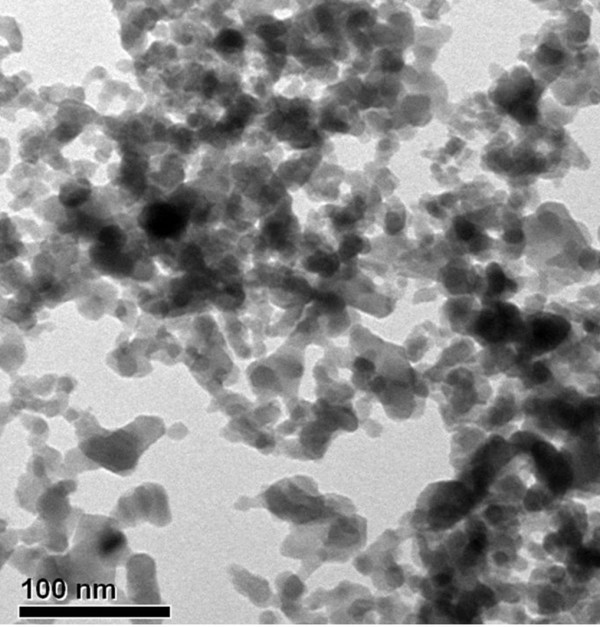
TEM image of Al_2_O_3_ additives.

**Figure 2:**
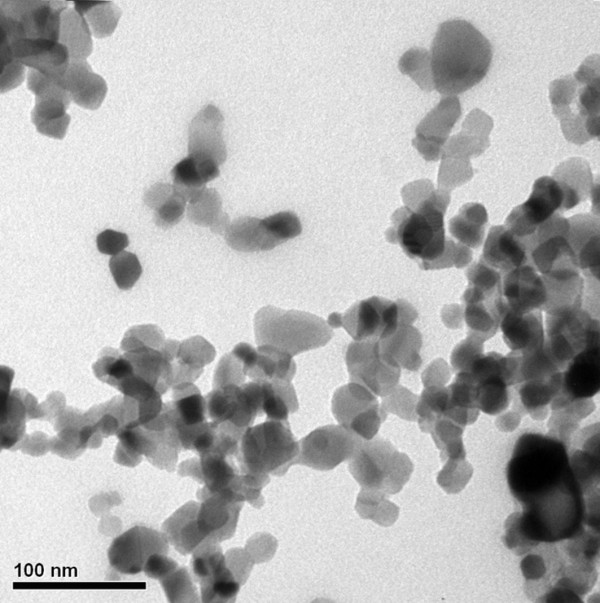
TEM image of TiO_2_ additives.

**Figure 3:**
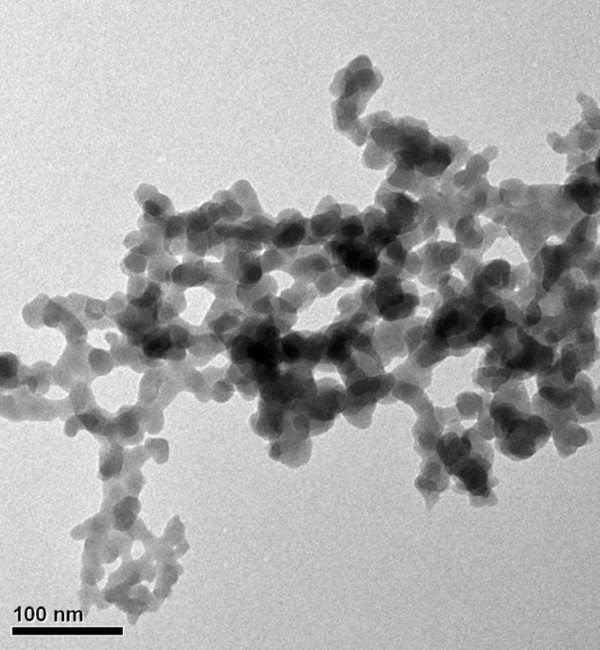
TEM image of SiO_2_ additives.

**Figure 4:**
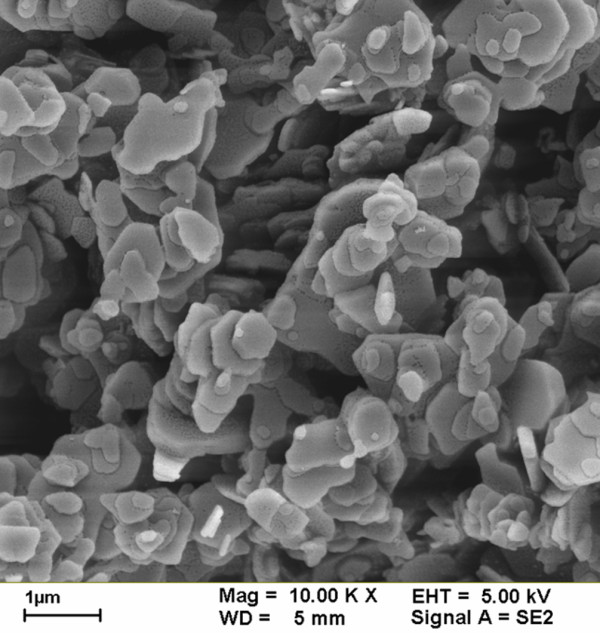
FE-SEM image of ZnO additives.

Fully refined paraffin (Choneye Pure Chemicals, Taipei, Taiwan) served as the base material in this study. Commercial Al_2_O_3_ (Al-13P, Yong-Zhen Technomaterial, Taipei, Taiwan), TiO_2_ (P-25, Degussa, Düsseldorf, Germany), SiO_2_ (Si-30P, Yong-Zhen Technomaterial), and ZnO (Zn-30, Yong-Zhen Technomaterial) were used to modify the paraffin. Figures 1, 2, 3, and 4 show the transmission electron microscope (TEM; H-7100, Hitachi, Tokyo, Japan) and field-emission scanning electron microscope (FE-SEM; LEO 1530, Zeiss, Oberkochen, Germany) images of Al_2_O_3_, TiO_2_, SiO_2_, and ZnO, respectively. The particle size distribution of Al_2_O_3_, TiO_2_, and SiO_2_ was approximately 20 to 30 nm, and the particle size distribution of ZnO was in the range of several hundred nanometers.

The additives were combined with paraffin to produce NEPCMs. NEPCMs produced by direct-synthesis method were used to disperse the additives in the paraffin into three weight fractions (1.0, 2.0, and 3.0 wt.%), which formed the experimental samples in this study. The liquid paraffin was then stirred continuously at 120°C using an electromagnetic stirrer/hot plate (PC420D, Corning Inc., Corning, NY, USA). The additives were added to the liquid paraffin divided by several times until the mixtures reached the desired concentration. The liquid NEPCMs were continuously homogenized for 40 min at 120°C using a high-speed homogenizer (T25 digital, IKA, Stanfen, Germany) at 6,000 rpm to evenly disperse the additives in the liquid paraffin. The liquid NEPCMs were then dispersed at 90°C for 1 h using an ultrasonic bath (D400H, TOHAMA, Hsinchu, Taiwan) to complete the synthesis of NEPCMs. Based on our past experience, the three dispersion devices have their own advantages and features. Interactive use of these machines can prevent the temperature of the devices from rising, and good dispersion of NEPCMs can be achieved in a shorter period.

The melted NEPCMs were poured into glass test tubes (30 cc) and allowed to cool and solidify. A spectrometer (BRC112E, B&W Tek, Newark, DE, USA) with an integrating sphere was used to test reflectivity of NEPCMs at 600 nm in the upper and lower parts (distance 12 cm) of the test tubes. A small difference in reflectivity between the upper and lower parts of the test tube indicates a high suspension performance of the additive suspended in the paraffin. In this study, the difference in reflectivity between the upper and lower parts of the test tube with an NEPCM must be less than 5% for the NEPCM to qualify as an experimental sample. The reflectivity difference of NEPCMs with Al_2_O_3_, TiO_2_, SiO_2_, and ZnO were 1.8%, 1.9%, 3.0%, and 3.9%, respectively.

### Experimental procedure and analysis

#### Heat conduction experiment

**Figure 5:**
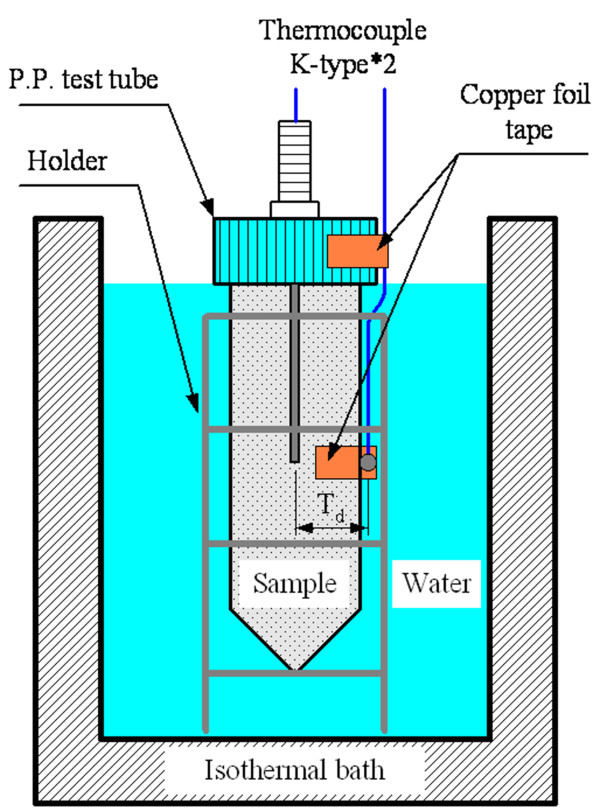
The test apparatus used for the heat conduction performance of NEPCMs.

This study investigates the effects of additives on temperature difference at a fixed location based on the paraffin to evaluate the heat conduction performance. The temperature difference of the experimental samples in steady state is lower, which represents the samples with higher heat conduction performance and thermal conductivity. Figure 5 shows the test apparatus used for the heat conduction performance of NEPCMs. The 40 g of melting, uniformly dispersing paraffin and NEPCMs was poured into a polypropylene test tube and then was cooled and solidified in an isothermal bath (P-20, YSC, Hsinchu, Taiwan) at 25°C to complete a unit of sample for the heat conduction experiment. Two thermocouples were installed on all test samples, and the temperature change was recorded by a data logger (TRM-20, TOHO, Kanagawa, Japan). Another isothermal bath (P-10, YSC) was stabilized to a test temperature (70°C, 75°C, 80°C, and 85°C), and the sample was then placed into the isothermal bath. The measurement time was 90 min for each sample, and the average temperature data during the last 10 min was referred to as a steady temperature. The steady-state temperature difference of the thermocouple was the steady-state temperature difference (*T*_d_) of the test samples.

#### DSC experiment

To select the proper PCMs, we must consider the suitable phase-change temperature and phase-change heat for the different temperatures of the heat sources and practical application. DSCs are often used to determine the phase-change temperature and phase-change heat of PCMs [23,25,31-33,37]. A DSC (Q20, TA, New Castle, DE, USA) with a vapor compression refrigeration cooling system (RCS40, TA) was used in the charging and discharging experiments in this study to assess the melting and solidification temperatures and the phase-change heat of the samples. The experimental temperature range was 25°C to 90°C at a fixed heating and cooling rate of 4.0°C/min. The calorimetric precision and temperature accuracy of the DSC were ±0.1% and ±0.1°C, respectively. The test samples were taken by a quantitative dropper after the NEPCMs were melted and stirred evenly using an electromagnetic stirrer/hot plate. Each sample was placed in an aluminum sample pan (Tzero pan, no: T100915) with a lid (Tzero hermetic lid, no: T100624), and the DSC experiment was conducted under high-purity nitrogen (5N) atmosphere. In this experiment, the sample's weight was controlled at 5.0 ± 1.5 mg in the sample pan using a precision electronic balance (XS-125A, Precisa, Dietikon, Switzerland) at a precision of 0.1 mg. The thermograms of the DSC charging and discharge experiments were analyzed by a computer software (Universal Analysis 2000, TA) and at a temperature ranging from 30°C to 70°C to calculate the phase-change latent heat for all the samples. Comparing the experimental results of DSC for NEPCMs with the paraffin under the same experimental parameters shows the effects of the additives on the melting and solidification temperatures and the heat of the paraffin.

#### Experimental data analysis

To compare the experimental data after adding the additives to paraffin (*D*_a_), all data obtained with the paraffin were gathered to form baseline values (*D*_p_). The experimental data obtained from the NEPCMs were compared with the baseline values. The differences before and after adding the additives to the paraffin are presented as percentage (*R*), and calculated as follows:

(1) $$R=\left(\frac{D_{a}-D_{p}}{D_{p}}\right)\times 100\%$$

## Results and discussion

**Figure 6:**
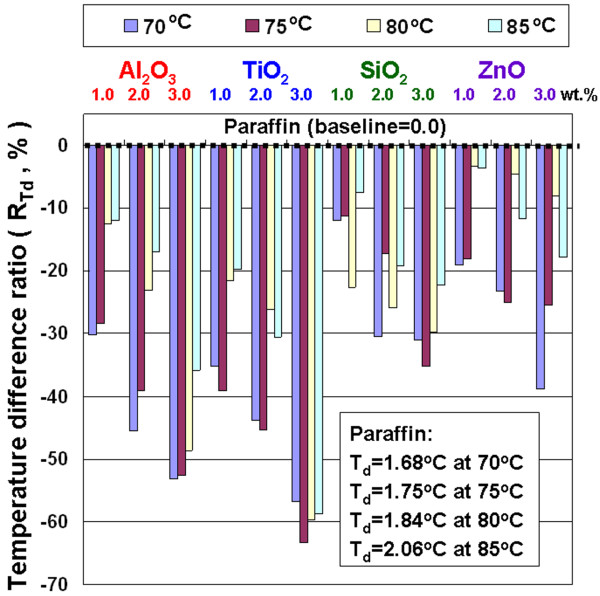
Temperature difference ratio of the NEPCMs.

In Figure 6, the *R*_*T*d_ of NEPCMs with Al_2_O_3_, TiO_2_, SiO_2_, and ZnO at both different concentrations (1.0, 2.0, and 3.0 wt.%) and test temperatures (70°C, 75°C, 80°C, and 85°C) shows a *T*_d_ lower than that of paraffin. The *T*_d_ of the experimental samples in steady state is lower, which represents the samples with higher heat conduction performance or thermal conductivity based on heat conduction theory. Experimental results show that adding these additives to the paraffin can really improve the thermal conduction performance (or thermal conductivity) of the paraffin, and adding TiO_2_ to the paraffin is the optimal selection. The NEPCMs can enhance the thermal conduction performance of the paraffin mainly because these additives have higher thermal conductivity as well as movement of additives in liquid paraffin to cause the quasi-convection phenomenon. Although the additives have similar thermal conductivity, the enhanced heat conduction performance of NEPCMs is obviously different mainly due to both the suspension performance of additives in paraffin and the combination performance between the additives and paraffin. The maximum decreased ratio of *T*_d_ is 63.3% at 75°C by adding TiO_2_ of 3.0 wt.% into the paraffin. Adding those additives to paraffin results in a higher heat conduction performance and allows the NEPCMs to response the rapid heat load changes in the charging and discharging process.

**Figure 7:**
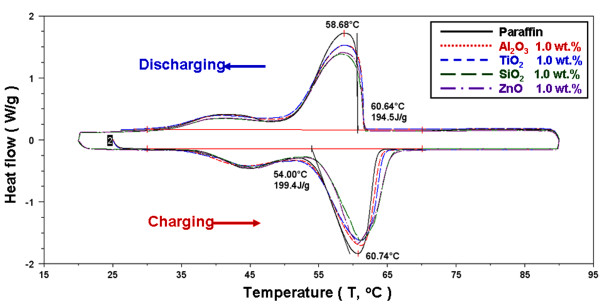
DSC experiment of endothermic and exothermic thermograms for paraffin and NEPCMs with a 1.0-wt.% concentration.

**Figure 8:**
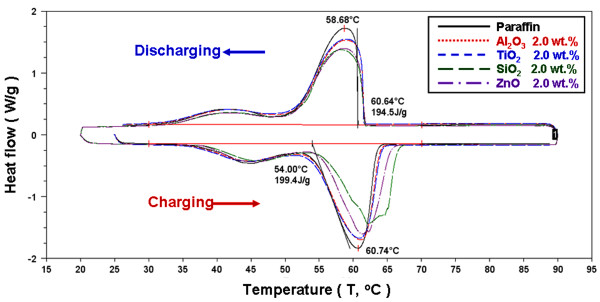
DSC experiment of endothermic and exothermic thermograms for paraffin and NEPCMs with a 2.0-wt.% concentration.

**Figure 9:**
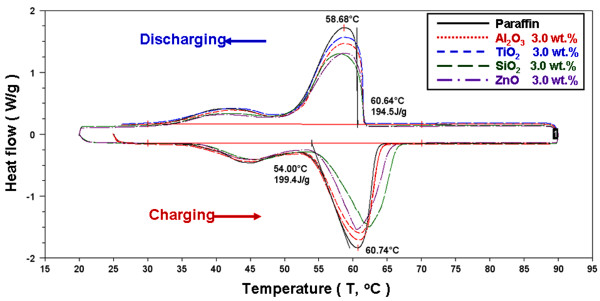
DSC experiment of endothermic and exothermic thermograms for paraffin and NEPCMs with a 3.0-wt.% concentration.

**Figure 10:**
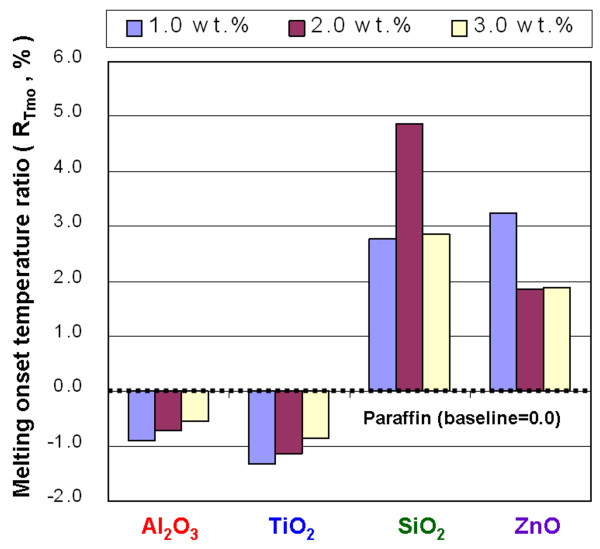
Melting onset temperature ratio of the NEPCMs.

**Figure 11:**
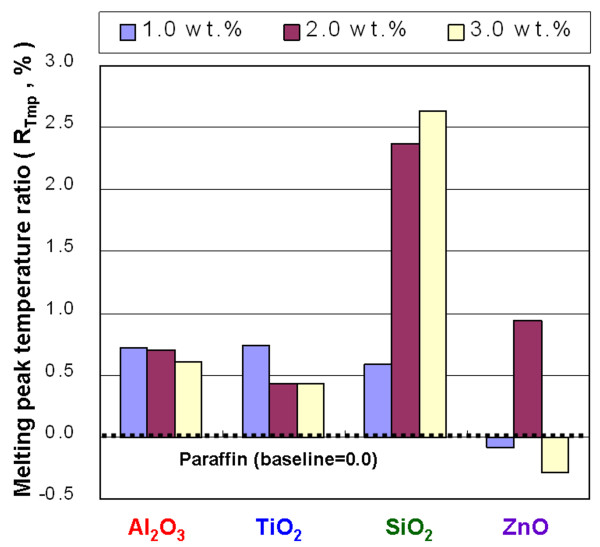
Melting peak temperature ratio of the NEPCMs.

**Figure 12:**
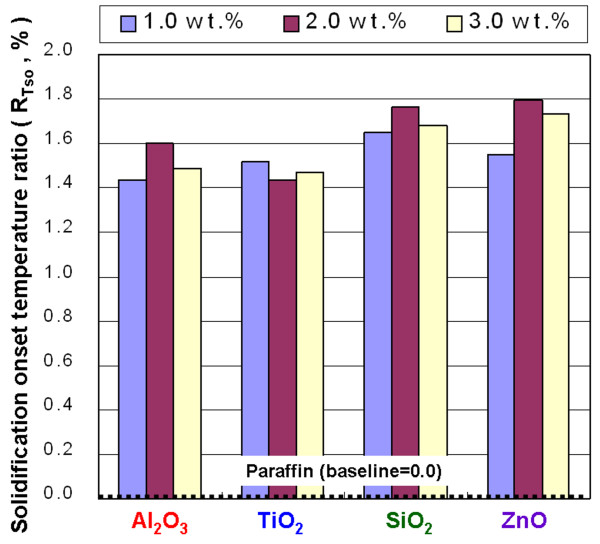
Solidification onset temperature ratio of the NEPCMs.

**Figure 13:**
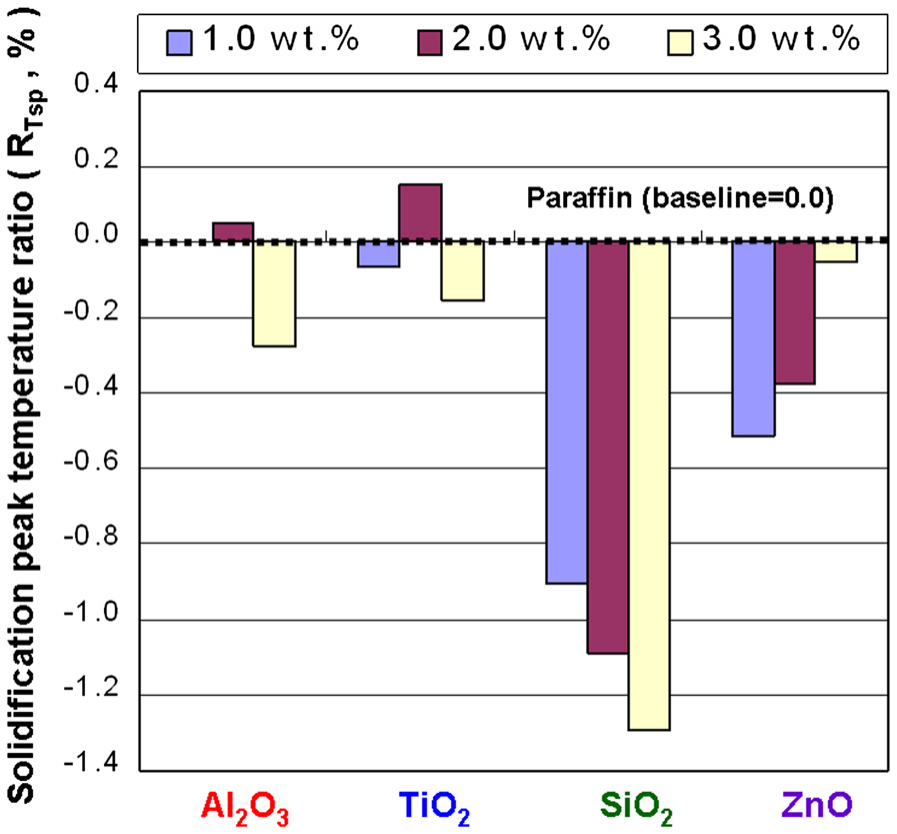
Solidification peak temperature ratio of the NEPCMs.

**Figure 14:**
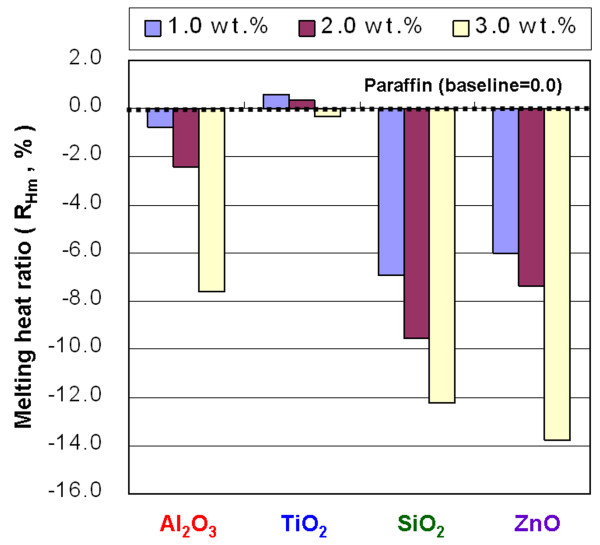
Melting heat ratio of the NEPCMs.

**Figure 15:**
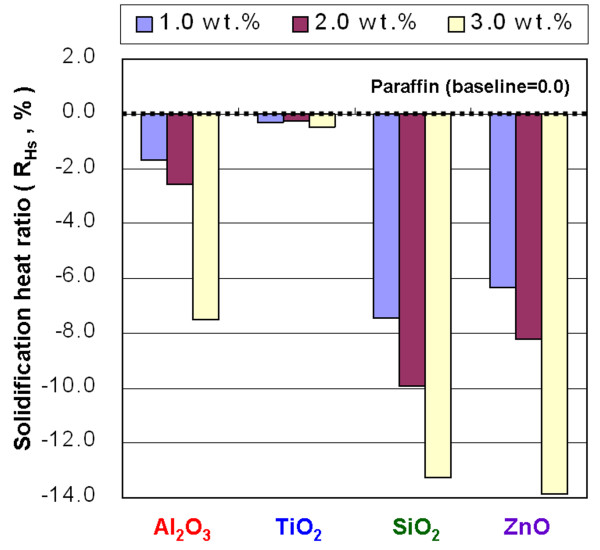
Solidification heat ratio of the NEPCMs.

Figures 7, 8, and 9 present the DSC thermograms of the NEPCMs with different additives and concentrations of additives. The phase-change latent heat for calculation ranges from 30°C to 70°C. As shown in the figures, during the melting and solidification process, the phase-change peak (*T*_p_) was 60.74°C and 58.68°C, the onset temperature (*T*_o_) was 54.00°C and 60.64°C, and the latent heat of phase change was 199.4 and 194.5 kJ/kg for paraffin, respectively. Because the paraffin was not a pure substance, its melting and solidification ranges were wider, compared to those of the pure substance. Furthermore, adding different additives at varying concentrations alters the endothermic and exothermic curve. The additives decreased the endothermic and exothermic peak and delayed the end of the melting point of the phase change. Table 1 shows the DSC experimental results of the NEPCMs for the melting and solidification processes with different additives and additive concentrations. To compare the effects of the charge and discharge characteristics of NEPCMs with different additives and additive concentrations, the data in Table 1 is further calculated as ratios by Equation 1 and are plotted in Figures 10, 11, 12, 13, 14, and 15. Figures 10, 11, 12, 13, 14, and 15 show the ratio of the melting onset temperature (*T*_mo_), melting peak temperature (*T*_mp_), solidification onset temperature (*T*_so_), solidification peak temperature (*T*_sp_), melting heat (*H*_m_), and solidification heat (*H*_s_) of the NEPCMs, respectively, as calculated by the experimental results. These experimental results show the differences between the NEPCMs and paraffin.

**Table 1:** Experimental results of DSC for NEPCMs

**Samples**	**Additive concentration (wt.%)**	**Process**	**Phase-change temperature (°C)**		**Latent heat (kJ/kg)**
			***T***_**s**_	***T***_**p**_	
Paraffin (P)	-	Charging	54.00	60.74	199.4
		Discharging	60.64	58.68	194.5
P + Al_2_O_3_	1.0	Charging	53.51	61.18	197.9
		Discharging	61.51	58.68	191.2
	2.0	Charging	53.61	61.17	194.6
		Discharging	61.61	58.71	189.5
	3.0	Charging	53.71	61.11	184.2
		Discharging	61.54	58.52	179.9
P + TiO_2_	1.0	Charging	53.28	61.19	200.6
		Discharging	61.56	58.64	193.9
	2.0	Charging	53.39	61.00	200.1
		Discharging	61.51	58.77	194.0
	3.0	Charging	53.54	61.00	198.7
		Discharging	61.53	58.59	193.6
P + SiO_2_	1.0	Charging	55.50	61.1	185.6
		Discharging	61.64	58.15	180.0
	2.0	Charging	56.62	62.18	180.4
		Discharging	61.71	58.04	175.2
	3.0	Charging	55.54	62.34	175.0
		Discharging	61.66	57.92	168.7
P + ZnO	1.0	Charging	55.75	60.69	187.4
		Discharging	61.58	58.38	182.2
	2.0	Charging	55.01	61.31	184.7
		Discharging	61.73	58.46	178.5
	3.0	Charging	55.02	60.57	171.9
		Discharging	61.69	58.65	167.5

Figure 10 shows the *R*_*T*mo_ of NEPCMs with Al_2_O_3_, TiO_2_, SiO_2_, and ZnO at concentrations of 1.0, 2.0, and 3.0 wt.%. A low *T*_mo_ in the NEPCMs allows the melting phase change to occur at a low temperature. In practical applications, this means that the melting phase change of NEPCMs can occur at a heat source with a relatively low temperature. Increasing the amount of Al_2_O_3_ and TiO_2_ in the paraffin reduces the *T*_mo_ of the paraffin, and increasing the amount of SiO_2_ and ZnO in the paraffin increases the *T*_mo_ of the paraffin. For NEPCMs with Al_2_O_3_ and TiO_2_ at concentrations of 1.0, 2.0, and 3.0 wt.%, the *T*_mo_ ratio decreased by 0.91%, 0.72%, and 0.54% and by 1.33%, 1.13%, and 0.85%, respectively. Adding the Al_2_O_3_ and TiO_2_ to the paraffin to form NEPCMs has better modification efficiency base on the test results of *T*_mo_, but increasing the concentration of SiO_2_ and ZnO will result in lower efficiency for lowering the *T*_mo_. Figure 11 shows the *R*_*T*mp_ ratio of NEPCMs with Al_2_O_3_, TiO_2_, SiO_2_, and ZnO at concentrations of 1.0, 2.0, and 3.0 wt.%. Increasing the amount of Al_2_O_3_, TiO_2_, and SiO_2_ can increase the *T*_mp_ of paraffin; however, increasing the amount of ZnO will not necessarily increase the *T*_mp_ of paraffin. The instability of the *T*_mp_ in the NEPCMs with ZnO is mainly a result of the larger particle size of ZnO, combination of the ZnO and paraffin, and poor suspension performance of ZnO in paraffin. Adding Al_2_O_3_, TiO_2_, and SiO_2_ to the paraffin resulted in a higher *T*_mp_ and allowed a melting phase-change peak of the paraffin to occur at higher temperatures.

In Figure 12, the *R*_*T*so_ of the NEPCMs with Al_2_O_3_, TiO_2_, SiO_2_, and ZnO at concentrations of 1.0, 2.0, and 3.0 wt.% shows a *T*_so_ higher than that of paraffin. The maximum enhanced ratio of *T*_so_ is 1.8% by adding ZnO of 2.0 wt.% into paraffin. Adding those additives to paraffin resulted in a higher *T*_so_ and allowed the solidification phase change of paraffin to occur at higher temperatures. This situation makes it possible to use a phase-change heat at higher temperatures to enhance the heat release rate of paraffin. Figure 13 shows the *R*_*T*sp_ of the NEPCMs with Al_2_O_3_, TiO_2_, SiO_2_, and ZnO at concentrations of 1.0, 2.0, and 3.0 wt.%. The effect of adding Al_2_O_3_ and TiO_2_ on *T*_sp_ is not obvious; however, adding SiO_2_ and ZnO to paraffin resulted in a lower *T*_sp_ and allowed a solidification phase-change peak of paraffin to occur at higher temperatures.

The *R*_*H*m_ of the NEPCMs with Al_2_O_3_, SiO_2_, and ZnO at concentrations of 1.0, 2.0, and 3.0 wt.% showed *H*_m_ lower than that of paraffin, but adding TiO_2_ to paraffin did not noticeably change the *H*_m_, compared to that of paraffin (Figure 14). The experimental results of *H*_s_ were similar to those for *H*_m_. Figure 15 shows that adding TiO_2_ to paraffin produced a minimal impact for *H*_s_. In the cases of both the *H*_m_ and *H*_s_, adding TiO_2_ decreased the phase-change heat of paraffin by less than 0.46%.

All experimental samples were tested for their suspension and combination performance with a spectrometer to confirm that the difference in reflectivity between the upper and lower parts of the test tube was less than 5%. During this process, the reflectivity differences of the NEPCMs with Al_2_O_3_ and TiO_2_ were below 2.0%, whereas the reflectivity differences of the NEPCMs with SiO_2_ and ZnO were above 3.0%. The NEPCMs with ZnO showed the lowest suspension performance, with a maximum reflectivity difference at 3.9%. Poor suspension performance of additives in paraffin cannot effectively enhance the heat conduction performance of NEPCMs due to sedimentation and uneven distribution of additives. Poor combination of additives with paraffin will produce a thicker interface layer to increase the interface thermal resistance between the paraffin and additives. This interface layer cannot contribute to the phase-change heat because phase change does not occur in the interface layer in the experimental temperature range, but it will reduce the unit volume of the thermal storage. In addition, the thicker thermal resistance layer also reduces the heat conduction performance of the NEPCMs. Furthermore, the thicker thermal resistance layer subjected to temperature changes has a relatively large expansion or contraction ratio, which will harm the combination of additives with paraffin and thereby reduce the thermal storage and heat conduction performance of the NEPCMs. Therefore, the suspension performances of additives, as well as the combination of additives and paraffin, are critical factors determining the heat conduction and thermal storage performance of NEPCMs.

In this study, the thermal conductivity of the additives was higher than that of paraffin. According to the calculations of thermal conductivity and temperature difference experiment, additives with a higher thermal conductivity than that of paraffin can enhance the thermal conductivity of the paraffin [43]. However, an additive in paraffin reduces the phase-change heat of the paraffin due to the fact that the additives do not cause phase change in the experimental temperature range. Such phenomena will reduce the unit volume of heat storage capacity and reduce the overall benefits of thermal storage. However, if the additives can reduce the onset temperature of the melting process or increase the onset temperature of the solidification process of NEPCMs, they will be able to enhance the usage range of phase-change heat of NEPCMs, thereby increasing the rate of charging and discharging more effectively in response to changes in the heat load due to phase-change heat having a large heat capacity. Therefore, in selecting the optimal additives, we must also consider the heat conduction performance and the range of phase-change temperature and phase-change heat. The experimental results show that adding TiO_2_ to paraffin can increase the range of phase-change temperatures and heat conduction performance, thereby increasing the temperature range of phase-change heat and the response rate of the thermal load that can be applied to heat storage. Furthermore, the NEPCMs with TiO_2_ have a minimum decreased ratio of phase-change heat of only 0.46% for maintaining an optimum heat storage capacity. Therefore, adding TiO_2_ to paraffin to enhance heat storage performance has notable potential for future applications.

## Conclusions

This study used a direct-synthesis method to prepare NEPCMs by adding varying concentrations of Al_2_O_3_, TiO_2_, SiO_2_, and ZnO nano-additives to paraffin. Through both the heat conduction and DSC experiments, we investigated the effects of varying the concentrations of the nano-additives on heat conduction performance and heat storage performance. Experimental results demonstrate that TiO_2_ is more effective than the other nano-additives in modifying the heat conduction and thermal storage performance of paraffin. Adding TiO_2_ can increase the heat conduction performance, reduce the melting onset temperature, and increase the solidification onset temperature of paraffin. This allows the application of phase-change heat to respond to rapid heat load changes and a wider temperature range, and the highest decreased ratio of phase-change heat is only 0.46%, compared to that of paraffin. Therefore, the use of TiO_2_ for enhancing the heat conduction and thermal storage performance of paraffin is a method that has great potential for future applications.

## Competing interests

The authors declare that they have no competing interests.

## Authors’ contributions

TPT designed the experiment and fabricated the samples. TPT and CCY carried out the measurements, analyzed the measurements, and wrote the paper. All authors read and approved the final manuscript.
